# Changes in the Urethral Artery After Experimental Subarachnoid Hemorrhage-Induced Injury of the Pudendal Ganglion and Onuf’s Nucleus Junction

**DOI:** 10.5152/eurasianjmed.2023.23202

**Published:** 2023-10-01

**Authors:** Mehmet Hakan Şahin

**Affiliations:** Department of Neurosurgery, University of Ataturk School of Medicine, Erzurum, Turkey

**Keywords:** Spinal subarachnoid hemorrhage, Onuf’s core, urethral vasospasm

## Abstract

**Objective:**

Onuf’s nucleus is an anatomical structure essential in the regulation of urogenital functions. Lumbosacral pathologies may cause changes in urogenital circulation due to Onuf’s nucleus injury; however, there is limited evidence corroborating the relationship between spinal cord injury and urethral artery changes.

**Materials and Methods:**

We used 23 sexually mature male rabbits—5 rabbits in the control group (GI), 5 rabbits in the sham group (GII), and 13 rabbits in the experimental group (GIII; received autologous blood transfusion into the T12–L1 subarachnoid space to induce subarachnoid hemorrhage (SAH). The GIII underwent a S1-3 laminectomy after 2 weeks and was decapitated. Histologic specimens were prepared to examine changes in Onuf’s nucleus, pudendal ganglion, and urethral arteries. The density of damaged neurons and vasospasm index (VSI) in the urethral artery were evaluated.

**Results:**

The mean density of damaged neurons (n/mm^3^) in Onuf’s nucleus and pudendal ganglia (S3) and the mean VSI of the 3 groups were as follows—GI: 6 ± 2 per mm^3^, 12 ± 4 per mm^3^, and 1.63 ± 0.25, respectively; GII: 27 ± 6 per mm^3^, 221 ± 62 per mm^3^, and 1.97 ± 0.36, respectively; GIII: 154 ± 41 per mm^3^, 1890 ± 541 per mm^3^, and 3.04 ± 0.95 (*P* < .05 each for GI/GII, GI/GIII, and GII/GIII). Neuronal damage criteria, such as cytoplasmic condensation and cytoplasmic halo formation, were more prominent in GIII.

**Conclusion:**

SAH can lead to ischemia of the Onuf’s nucleus–pudendal nerve structures due to urethral artery spasm, resulting in urogenital complications.

**Main Points**

- Spinal subarachnoid hemorrhage (SAH) in the thoracosacral area may affect the urogenital system.
- Urethral artery vasospasm may be observed after spinal SAH.
- Damage to Onuf’s nucleus and pudendal ganglion may occur secondary to arterial vasospasm.
- Damage to neural mechanisms involving the urogenital system may impair spermatogenesis.

## Introduction

A combination of various sympathetic, parasympathetic, and somatosensitive nerves are involved in the functioning of the urogenital tissues. In a human male, vasodilatory parasympathetic impulses originate from Onuf’s nucleus located in the spinal cord, whereas vasoconstrictor sympathetic nerves are connected to the sympathetic region of the spinal cord at the lumbar (L) 1 and 2 (L1-L2) levels.^1^ Furthermore, somatosensory neurofibers originate from the dorsal root ganglia of L5 and first sacral (S1) levels. Human sexual function is also controlled by sacral parasympathetic and thoracolumbar sympathetic connections.^2^ Parasympathetic innervation from S2-S4 controls erection, and sympathetic innervation of the pelvic plexus prevents retrograde ejaculation in the male genital organs.^3^ Consequently, cordotomy may cause micturition and sexual and reproductive problems in male patients^4^ because sympathetic nerves, in the absence of parasympathetic innervation, may cause vasospasm in penile arteries.^1^

Subarachnoid hemorrhage (SAH) is an important condition that can affect the cranial and spinal regions,^5^ and several studies are ongoing to understand this condition.^6^ Spinal SAH is known to cause vasospasm in the arteries supplying the dorsal root ganglion (DRG), resulting in severe ischemic damage to the DRG and ultimately to the spinal cord.^7^ Parasympathetic innervation of testicular tissues is obtained from the sacral parasympathetic network, of which the Onuf’s nucleus is an important component; therefore, a disruption of this network following spinal SAH may cause both testicular denervation damage and abnormal spermatogenesis due to testicular artery spasm. There are reports of ischemic spinal cord injury or spinal cord damage after spinal SAH affecting spermatogenesis in humans^8^ and rats,^9^ especially erection and ejaculation functionally and spermatogenesis morphologically. Several effects, such as impaired erection, ejaculation, spermatogenesis, decreased sperm viability and motility, and morphologic abnormalities,^10^ are reported in these situations, which highlight the importance of Onuf’s nucleus in infertility. In this regard, the present study aimed to investigate the effects of injury to the Onuf’s nucleus on organs related to sexual functions in rats with spinal SAH.

## Materials and Methods

### Experimental Design

This study was conducted in accordance with national and international guidelines for the use of experimental animals. The experimental protocol was reviewed and approved by local and official committees for animal care and use (approval number: E-42190979-000-2200226662). Twenty-three sexually mature male rabbits (obtained from the Local Experimental Laboratories; bodyweight = 3200-3700 g; housed at room temperature 15-18°C) were used for all experiments in this study. Five rabbits were placed in the control group (GI), 5 rabbits in the sham group (GII), and 13 rabbits in the experimental group (GIII). Each rabbit was given subcutaneous ketamine hydrochloride (Ketalar® 50; 25 mg/kg), lidocaine hydrochloride (Aritlmal^®^; 15 mg/kg), and acepromazine (Plegicil®; 1 mg/kg) to induce general anesthesia.

To induce the SAH in the sham and experimental groups, the injection point in the subarachnoid space was chosen posteriorly at the level of the 12th thoracic (T12)-L1 level. The sham group was injected with 0.7 mL saline, whereas the experimental group was injected with 0.7 mL autologous blood. After the injection, the incision areas were closed with sutures. Antibiotics and analgesics were not given to prevent any effect on the study results.

Two weeks later, we cleaned (with antiseptic) and shaved the lumbosacral regions of all animals, and an S1-3 laminectomy was performed under general anesthesia. The spinal cord, pudendal ganglia, and penile tissues were removed from the S3 level and fixed in a 10% formalin solution. The tissues were placed in paraffin blocks, sectioned into 5 μm sections, and stained with hematoxylin and eosin stain. The serologic method was used to determine the neuron density of Onuf’s nucleus and pudendal ganglia.^8^

### Penile Tissue Examinations

Ten consecutive midline coronal sections of all penile tissues were examined by routine histopathologic methods. Each paired consecutive section of penile specimens was examined by stereologic methods, and the vasospasm index (VSI) values of the urethral arteries were determined.^11^ For measuring VSI, the outer diameter of the vessels was marked as 2R and the inner diameter of the vessels as 2r. For obtaining VSI values, the outer surface value was divided by the lumen surface value using the following formula: (πR^2^ − πr^2^/πr^2^) = (R^2^ − r^2^/r^2^).^12^

### Statistical Analysis

All analyses were performed using Statistical Package of the Social Sciences Statistics software, version 20.0 (IBM SPSS Corp.; Armonk, NY, USA). The normality of the distribution of the data was examined using the Shapiro–Wilk test. Accordingly, ANOVA, or the Kruskal–Wallis tests, followed by post hoc testing, were used for group-wise comparisons. A *P*-value of <.05 was considered statistically significant.

## Results

**Histopathologic** **Findings of the Spinal cord,** **Pudendal** **nerve, and Penile Tissue Specimens**

Morphologically, the spinal cord was edematous, and black to dark brown blood clots were seen scattered in the subarachnoid spaces. The testes were reduced in size and volume and appeared softened and shrunken. After SAH, arterial vasospasm, damaged nerve roots and dorsal root ganglia, apoptotic nerve stem cells, and DRG neurons were observed.

**Figure 1. A-D.**
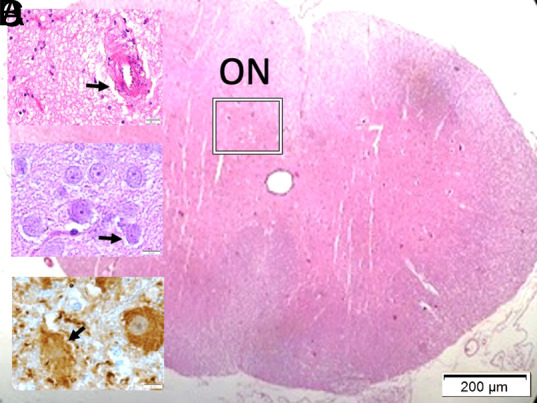
Coronal section of the spinal cord at the S2 level showing the location of Onuf’s nucleus on the gray matter in the anteromedial part (A); intramedullary spinal cord artery (LM, H&E, ×4/A; ×40/BC; GFAP, ×40/D) in a subject animal from Group III (B); normal neurons in Onuf’s nucleus seen in a control rabbit are marked with arrows. H&E, hematoxylin and eosin; LM, light microscopy.

**Figure 2. A-B.**
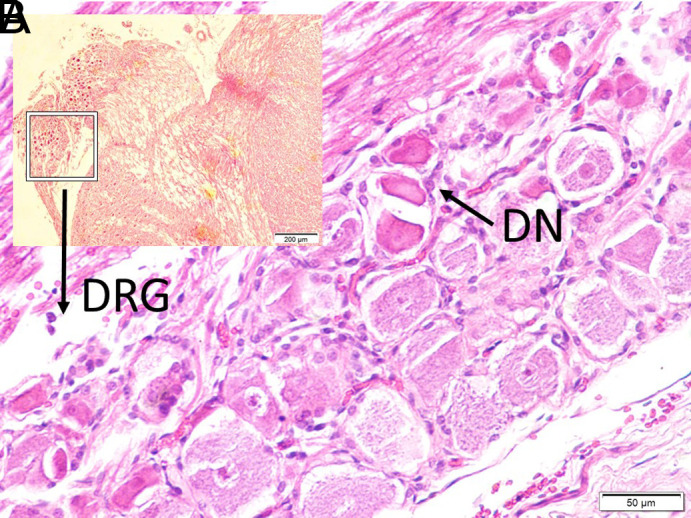
Coronal section of the spinal cord at the S2 level showing normal and degenerated neurons in Onuf’s nucleus in a subject belonging to group 3 (LM, H&E, ×4/A; ×20/B). H&E, hematoxylin and eosin; LM, light microscopy.

**Figure 3. A-C.**
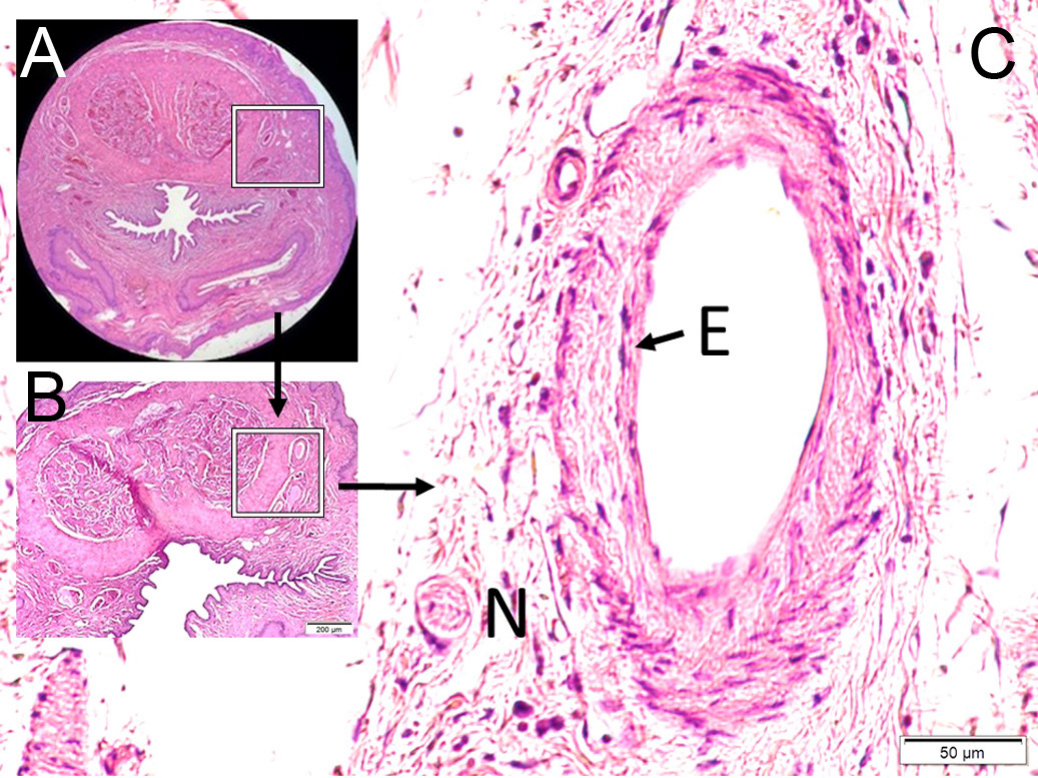
Coronal section of penile tissue at mid-level of the urethra and periurethral arteries (A); magnified (B). (C) A control animal showing the endothelium of the urethral artery (E) and a branch of the pudendal nerve (N) (LM, H&E, ×4/A; ×10/B and ×20/C). H&E, hematoxylin and eosin; LM, light microscopy.

**Figure 4. A-C.**
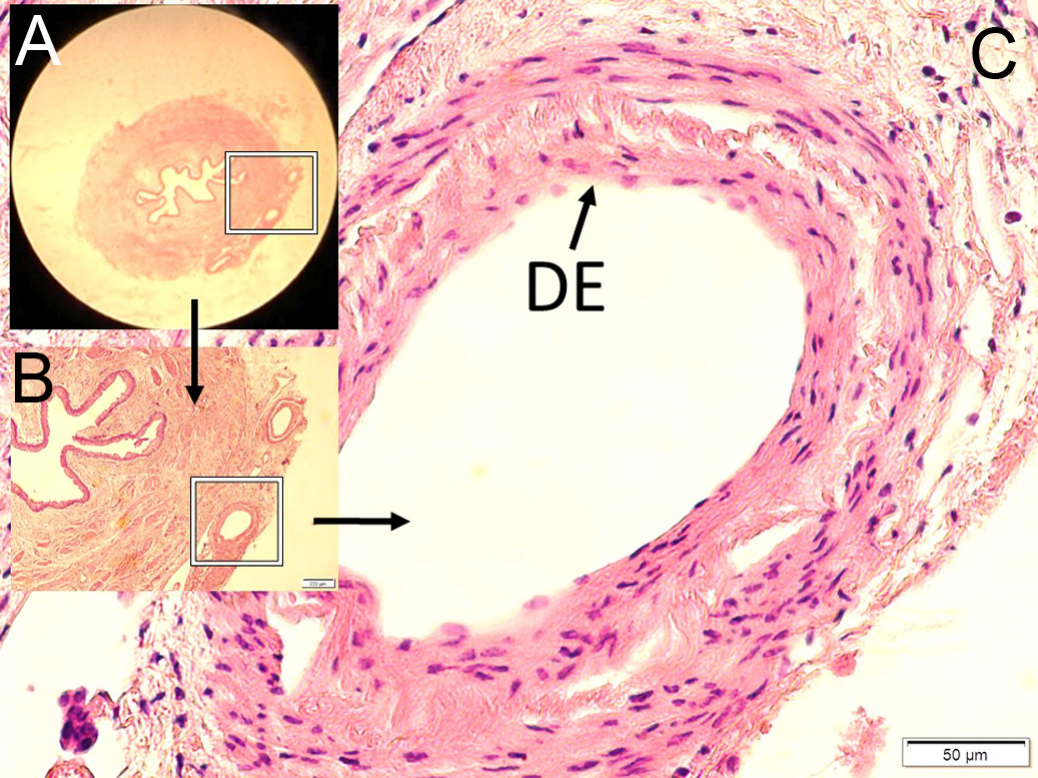
Coronal section of penile tissue at mid-level of the urethra and periurethral arteries (A); enlarged view (B). (C) A mildly contracted urethral artery with moderately degenerated endothelium (DE) in an animal from the sham group (LM, H&E, ×4/A; ×10/B and ×20/C). H&E, hematoxylin and eosin; LM, light microscopy.

**Figure 5. A-C.**
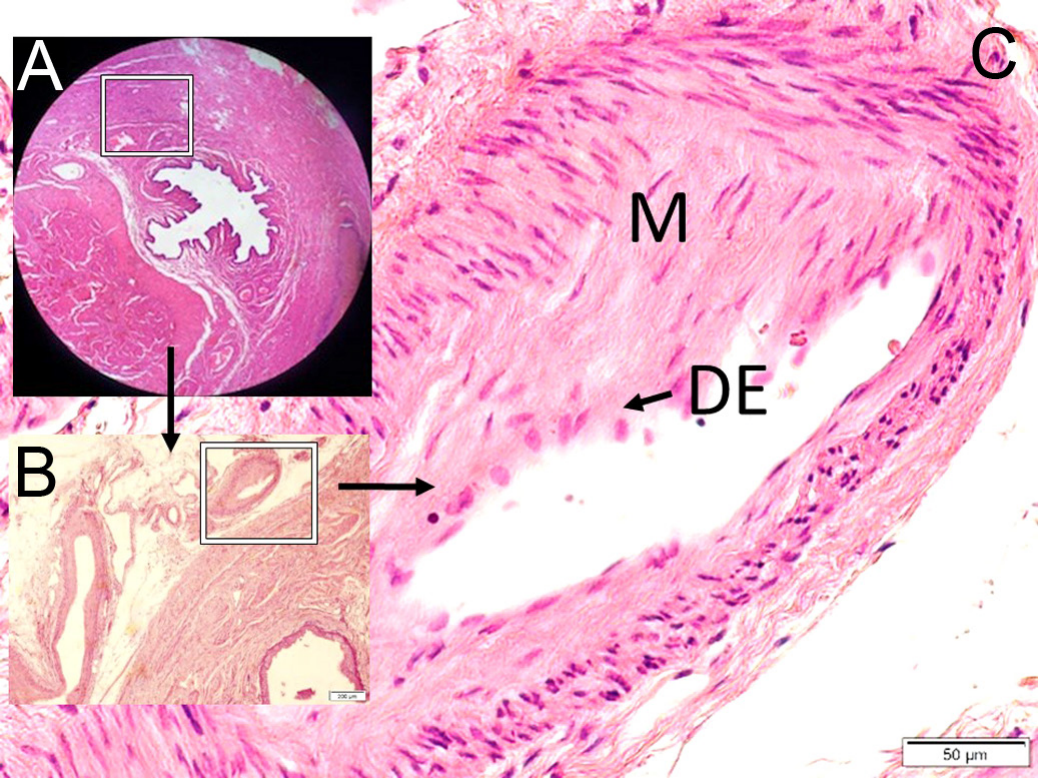
Coronal section of penile tissue at mid-level of the urethra and periurethral arteries (A); enlarged view (B). (C) A severely contracted urethral artery with severely degenerated endothelium (DE) in an animal from the experimental group (LM, H&E, ×4/A; ×10/B and ×20/C). H&E, hematoxylin and eosin; LM, light microscopy.

In the control group, coronal sections of the spinal cord at the S2 level showed healthy neuronal tissue, Onuf’s nucleus located anteromedially in the gray matter, and its intramedullary artery (Figure 1). In contrast, coronal sections of the S2 level in the experimental group showed normal and damaged neurons in the pudendal nerve root/ganglia (DRG/A) and pudendal ganglia (Figure 2). Additionally, vascular wall damage, endothelial necrosis, and thrombus were observed in the spinal radicular arteries of the experimental group animals (Figure 3). Regarding penile tissue examination, a specimen from the sham group showed a urethral artery with moderately degenerated endothelium (Figure 4), whereas in the experimental group, a destructed urethral artery with severely degenerated endothelium was observed (Figure 5).

Neuronal shrinkage, cellular angulation, cytoplasmic halo, and cytoplasmic condensation, which are the criteria for neuronal damage, were more prominent in the experimental group compared to the other groups.

The mean density of damaged neurons (n/mm^3^) in Onuf’s nucleus and pudendal ganglia (S3) and the mean VSI of the 3 groups were as follows: GI: 6 ± 2 per mm^3^, 12 ± 4 per mm^3^, and 1.63 ± 0.25, respectively; GII: 27 ± 6 per mm^3^, 221 ± 62 per mm^3^, and 1.97 ± 0.36, respectively; GIII: 154 ± 41 per mm^3^, 1890 ± 541 per mm^3^, and 3.04 ± 0.95. There was a statistically significant difference between GI and GII (*P* < .005), GI and GIII (*P* < .000), and GII and GIII (*P* < .000).

## Discussion

Male genital organs have bud-shaped structures in the penile tissues that modulate the sensation of voiding and orgasm; these structures are innervated by the pudendal nerves, whose fibers originate in Onuf’s nucleus.^12^ Consequently, connection disorders of the pudendal nerve, Onuf’s nucleus, and urethral taste rosea damaged after spinal SAH may lead to the development of dysorgasmia or anorgasmia.^13^ The Adamkiewicz artery plays an important role in the supply of the pudendal nerve roots and the conus medullaris, and urinary retention may develop secondary to ischemic events following the spasm of this artery.^6^ The resultant ischemia of Onuf’s nucleus should be considered among the causes of vasospasm in the mesenteric artery region after SAH.^11^ Furthermore, this secondary ischemic damage to Onuf’s nucleus after spinal SAH is known to adversely affect sperm count.^8^

The male genital organs receive sacral parasympathetic fibers via the pudendal nerves, and pudendal nerve damage is a known cause of chronic penile numbness and erectile dysfunction.^12^ The spinal sympathetic fibers and parasympathetic neurons of Onuf’s nucleus are 2 important neural structures innervating both the bulbourethral gland and the corpus spongiosum. Preganglionic impulses originate from the L1-L2 pelvic nerve ganglia; the main penile nerve axons originate from the L5-S1 DRG; and sympathetic axons in the abdominopelvic chain.^1^ Parasympathetic penile nerves come from Onuf’s nucleus in the parasympathetic area of the spinal cord; sympathetic nerves come from L1-2 in the sympathetic region of the spinal cord; and somatosensory neurons of the DRG come from L5-S1.^1^ In our study, we found that the degree of spasm in the urethral arteries and the level of vascular degeneration increased as the degree of neuronal damage in Onuf’s nucleus and pudendal network increased.

Like all motor and sensory processes, sexual function also requires a healthy spinal cord. This is achieved by the coordination of the sacral parasympathetic nerves and the thoracolumbar sympathetic nerve connection.^14^ Considering that sexual senses are regulated by an organization between the taste bud-like structures and the pudendal nerve, penile organ surgery or damage can disrupt this organization, which may negatively affect sexual function and desire.^2^ At present, this is a corrigible theory and requires confirmation by further studies; however, it may provide a different perspective on the causes and treatment of sexual pathologies.^13^ The urethral taste buds are also innervated by the neural network controlled by the Onuf’s nucleus–pudendal nerve and supplied by the urethral arteries, and pathologies in this neurovascular network may lead to degeneration of the taste buds and sexual function problems. In patients sustaining quadriplegia after spinal cord injury, infertility is often observed due to impaired spermatogenesis resulting from damaged neural connections to the testes.^15^ In the present study, post-SAH changes were seen in histopathological examination of both spinal cord and penile tissue specimens—arterial occlusion due to clots, degenerated nerve roots, degenerated DRG, apoptotic nerve stem cells around the spinal cord, neuronal damage in Onuf’s nucleus and pudendal ganglia, and an increase in testicular arteries in favor of vasospasm.

A problem with the connection between the Onuf’s nucleus–pudendal nerve–testis network can affect sperm quantity through neuronal damage controlling spermatogenesis. Oxygenation is important for the proper functioning of all tissues, especially neurons.^16^ Vasospasm, an important complication after SAH, may cause ischemia, which can particularly cause degeneration of Onuf’s nucleus.^17^ Linsenmeyer et al observed that testicular blood flow decreased after spinal cord injury;^18^ subsequent examinations showed that semen quality was also reduced in their patients. It has also been reported that severe spinal cord injury from T9 to L1 levels inhibits spermatogenesis,^19^ suggesting the role of sacral parasympathetic nerves and thoracolumbar sympathetic nerve connections for spermatogenesis. These findings corroborate our results that testicular artery spasm due to Onuf’s nucleus degeneration following spinal SAH may affect both sperm quality and sexual functions.

This study highlights the role Onuf’s nucleus and urethral connections may have on micturition and sexual function, and affection of this neural network following SAH or any spinal trauma may be a pathophysiologic factor leading to urethral dysfunction. Our results shed light on the relatively unknown Onuf’s nucleus–pudendal nerve–urethra connection and its impact on urogenital dysfunction.

This study provides novel information on the signs of vasospasm of the urethral arteries after spinal SAH. We found that spinal SAH can lead to significant urethral artery spasms and impair neuronal transmission through Onuf’s nucleus. Further studies with specific histological staining and longer follow-up are needed to better understand this issue.

### Limitations

First, we experimentally induced spinal cord injury in an animal model; it was not possible to verify these findings in vivo in humans. Second, the sample size of our study may not be sufficient. As mentioned in the study, the autonomic innervation of the testicles is managed through thoracolumbar sympathetic and sacral parasympathetic nerves. We know that disruption of sacral spinal cord circuits after spinal cord injury causes changes in testicular function. Based on the results of the present study, we believe arterial occlusion and decreased blood flow to be a possible causal mechanism for the decreased mean sperm count following spinal SAH. In this study, we only examined the sacral parasympathetic network through Onuf’s nucleus. Future studies should evaluate the effect of sympathetic fibers on spermatogenesis.
